# Effects of Lipolysis-Stimulated Lipoprotein Receptor on Tight Junctions of Pancreatic Ductal Epithelial Cells in Hypertriglyceridemic Acute Pancreatitis

**DOI:** 10.1155/2022/4234186

**Published:** 2022-04-14

**Authors:** Jie Wang, Mengbin Qin, Qing Wu, Huiying Yang, Biwei Wei, Jinlian Xie, Yingying Qin, Zhihai Liang, Jiean Huang

**Affiliations:** ^1^Department of Gastroenterology, The Second Affiliated Hospital of Guangxi Medical University, Nanning, China; ^2^Department of Gastroenterology, The First Affiliated Hospital of Guangxi Medical University, Nanning, China

## Abstract

**Objective:**

We investigated the effects of lipolysis-stimulated lipoprotein receptor (LSR) on the tight junctions (TJs) of pancreatic ductal epithelial cells (PDECs) in hypertriglyceridemic acute pancreatitis (HTGAP).

**Methods:**

Sprague-Dawley rats were fed standard rat chow or a high-fat diet and injected with sodium taurocholate to obtain normal and HTGAP rats, respectively. Serum triglyceride (TG) levels, pathological changes, TJ proteins in the pancreas, and TJ ultrastructure of PDECs were assessed. LSR overexpression (OE) and knockdown (KD) HPDE6-C7 models were designed and cultured in a high-fat environment. Protein levels were quantified by Western blotting. Cell monolayer permeability was detected using FITC-Dextran.

**Results:**

Serum TG concentration and pancreatic scores were higher in the HTGAP group than in the normal group. Among the TJ proteins, LSR protein expression was significantly lower in the HTGAP group than in the acute pancreatitis (AP) group. Tricellulin (TRIC) expression in the pancreatic ductal epithelia was higher in the HTGAP group than in the AP group. The HTGAP group had lower TJ protein levels, wider intercellular space, and widespread cellular necrosis with disappearance of cell junction structures. In the cell study, TJ proteins were downregulated and the cellular barrier was impaired by palmitic acid (PA), which was reversed by LSR-OE, whereas LSR-KD downregulated the TJ proteins and aggravated PA-induced cellular barrier impairment.

**Conclusions:**

Hypertriglyceridemia downregulates the TJ proteins in PDECs, which may impair the pancreatic ductal mucosal barrier function. LSR regulation can change the effects of HTG on cellular barrier function by upregulating the TJ proteins.

## 1. Introduction

Hypertriglyceridemia (HTG) is the third most common etiology of acute pancreatitis (AP), following gallstones and alcohol. Patients with HTG suffer from severe AP (SAP) and organ failure [1]. To date, the pathogenesis of hypertriglyceridemic AP (HTGAP) remains unclear. Therefore, it is crucial to clarify the pathogenesis of HTGAP and propose more effective prevention and treatment measures to improve the prognosis of HTGAP.

Pancreatic ductal epithelial cells (PDECs) and their secreted mucus layer constitute the pancreatic ductal mucosal barrier (PDMB), which can not only prevent the entry of bile and trypsin into the pancreatic parenchyma and the reflux of HCO_3_^−^ present in the pancreatic juice into the blood but also regulate the production of prosecretase in acinar cells [2]. The impairment of the PDMB is an important early event in the pathogenesis of AP. The tight junctions (TJs) in PDECs are essential ultrastructures that maintain the structural and functional integrity of epithelial cells. In addition, they play an important role in maintaining the functions of PDMB. Animal studies show that transient high pressure in the pancreatic duct caused by the retrograde biliary-pancreatic ductal injection of normal saline can destroy the TJ structures in pancreatic tissues and lead to pancreatitis [3]. Furthermore, other studies have found that the long-term stimulation of a large number of free fatty acids (FFAs) can lead to epithelial cell contraction, decreased intercellular TJs, and easy infiltration of lipids, leading to damage of the PDMB [4]. Hence, in addition to the acute inflammatory injury of pancreatic tissue caused by HTG, a long-term HTG environment may lead to abnormal ultrastructural changes in pancreatic tissues, especially in the PDMB, which can induce AP.

The TJ is of two types: bicellular tight junction (bTJ) and tricellular tight junction (tTJ). The bTJ is mainly composed of occludin and claudin protein families. It controls the flow of macromolecules and ions into and out of cells. It plays an important role in the inflammation of the digestive tract, respiratory tract, and central nervous system, as well as in the invasion and metastasis of tumors [5]. Moreover, increased occludin and claudin-5 expression in PDECs and acinar cells has been found to reduce pancreatic tissue damage in an AP rat model [6]. The tTJ is composed of tricellulin (TRIC) and angulin protein components. It plays a key role in maintaining cell barrier function and morphology of various tissues, composed of cells [7]. Lipolysis-stimulated lipoprotein receptor (LSR) is the core protein of the angulin protein family. It is located at the center of the tTJ and plays an essential role in regulating the function and morphology of tTJs by supplementing the TRIC expression in junctions [8]. It stably expresses in tTJs when the cell density is high and shifts to bTJs when the cell density is low. Furthermore, it promotes the expression of F-actin to maintain the TJ stability [9]. Therefore, LSR is not only the main supplement of TRIC but also one of the key proteins in regulating TJs. However, the role of LSR in TJs in the HTGAP is poorly understood, and abnormal ultrastructural changes of PDMB in pancreatic tissues have not been reported.

The obesity pandemic may be a likely contributor to the increasing incidence and worsening severity of AP [10, 11]. Obesity is associated with increased visceral fat in or around the pancreas, which can worsen AP outcomes and increase the risk of HTG both preceding and during an attack of pancreatitis [12, 13]. The mechanisms by which obesity may exacerbate pancreatitis include unsaturated fatty acid (UFA) toxicity to pancreatic cells, increase in the levels of inflammatory mediators, and reduced ATP levels via the inhibition of mitochondrial complexes [10].

In this study, we explored the effects of LSR on TJs in HTGAP using a rat model and a human pancreatic ductal epithelial cell line (HPDE6-C7) to provide a theoretical basis for the pathogenesis of HTGAP.

## 2. Materials and Methods

### 2.1. Animal and Experimental Design

**Figure 1:**
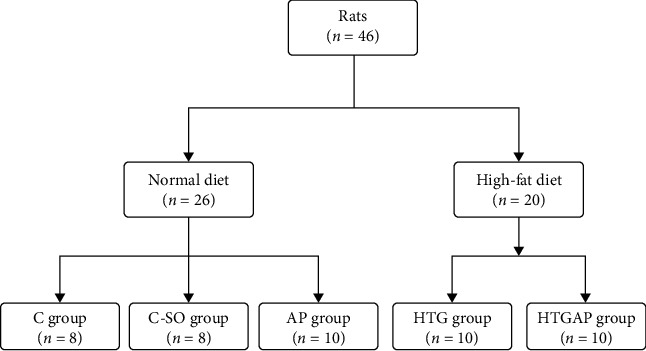
Animal experimental grouping method.

In this study (Figure 1), we included 46 male, four-week-old Sprague-Dawley rats from the Laboratory Animal Center of Guangxi Medical University (Nanning, China). These rats were housed at 24°C with a 12/12 h light-dark cycle. They were given free access to water and chow. The study was conducted in accordance with the recommendations of the Animal Protection Guidelines and approved by the local animal ethics committee. The protocol was approved by the Animal Care & Welfare Committee of Guangxi Medical University (No. 202005011).

Rats were randomly divided into five groups: normal diet control group (C group, *n* = 8), normal diet sham-operated group (SO group, *n* = 8), normal diet AP group (AP group, *n* = 10), high-fat diet control group (HTG group, *n* = 10), and high-fat diet AP group (HTGAP group, *n* = 10). Rats in the high-fat diet groups (HTG and HTGAP) were fed with a high-fat diet (82.8% standard rat chow+15% saturated animal fat+2% cholesterol+0.2% sodium cholate), and those in the normal diet group (C, SO, and AP) were given standard rat chow.

After 4 weeks of feeding, the rats were fasted for 12 h prior to surgical procedures. The AP rat model was created by retrograde injection of 4% sodium taurocholate (TCI, Tokyo, Japan) into the biliary-pancreatic duct at a speed of 1 mL/min/kg. The pancreases of rats in the SO group were flipped and returned to the original position through laparotomy. Rats were anesthetized with intraperitoneal injection of 40 mg/kg pentobarbital sodium. Rats were euthanized by bloodletting at 24 h after modeling. The blood samples were collected from the abdominal aorta and centrifuged for 15 min at 2000 rpm, and the serum was conserved at -80°C. One part of the pancreas was immediately frozen in liquid nitrogen and conserved at -80°C for further analyses. The rest of the pancreatic tissue was fixed using 10% formalin, embedded in paraffin, and used for morphologic assessment by hematoxylin and eosin (H&E) staining.

### 2.2. Serum Triglyceride Assay

Triglycerides in the serum collected from the rats were detected using an Automatic Biochemistry Analyzer (Hitachi Corporation, Japan) following standard procedures.

### 2.3. Histopathological Analysis

The pancreatic specimens of rats were sectioned at 3 *μ*m and stained with H&E staining. Two professional specialists, who were blinded to the experiments, morphologically evaluated the specimens under a light microscope. Changes in pancreatic histopathology were measured and classified in accordance with the study by van Laethem et al. [14].

### 2.4. Immunohistochemical Analysis

Tissue sections collected from the pancreases of rats were deparaffinized, and antigens were retrieved through high pressure cooking with citrate buffer (pH 6.0) for 5 min. Then, these tissue sections underwent gradient cooling until room temperature and extensive washing in phosphate-buffered saline (PBS, pH 7.4). Next, they were quenched with 3% hydrogen peroxide for 30 min, blocked with 3% bovine serum albumin at room temperature for 15 min, and incubated overnight at 4°C with 1 : 100 dilutions of the primary antibody against LSR (1 : 100 dilution, Sigma, USA), TRIC (1 : 100 dilution, Sigma, USA), claudin-1 (1 : 100 dilution, ABclonal, Wuhan, China), ZO-1 (1 : 100 dilution, Proteintech, Wuhan, China), occludin (1 : 100 dilution, Proteintech, Wuhan, China), and claudin-1 (1 : 50 dilution, ABclonal, Wuhan, China). The next day, the tissue sections were washed and incubated with a horseradish peroxidase-labeled secondary antibody (ZSGB-BIO, Beijing, China) for 15 min at room temperature. The slides were placed in PBS, washed three times on a decolorizing shaker for 5 min per wash, and visualized with diaminobenzidine (50 *μ*L per slide; DAB, ZSGB-BIO, Beijing, China). The pathology image analysis system (Leica DMR+Q550, Germany) was used for the visualization of these slides. The average of optical density (AOD) of images was measured using Image-Pro Plus 6.0 (Media Cybernetics, USA).

### 2.5. Transmission Electron Microscopy

In this study, intercellular TJs were observed using transmission electronic microscopy. The biliary-pancreatic ducts of rats were isolated, washed with PBS, and quickly placed in 3% glutaraldehyde fixative solution. It was then placed in a refrigerator at 4°C for more than 2 h. Next, tissue sections were washed and fixed with 1% osmium acid fixative for 2 h at 4°C. Then, they underwent dehydration, soaking, embedding, and polymerization. The sections were stained with methyl blue+alkaline fuchsin and sectioned using an ultrathin microtome (LK-UC7). The changes in TJs between the biliary and pancreatic ductal epithelia were observed using transmission electronic microscopy (H-7650, Hitachi Ltd., Tokyo, Japan).

### 2.6. Cell Culture, Infection, and Treatment

The normal human pancreatic ductal epithelial cell line (HPDE6-C7) was purchased from the Beijing Beinachuanglian Biotechnology Research Institute (Beijing, China) and cultured in an appropriate density with the recommended medium, supplemented with 10% fetal bovine serum in a humidified incubator containing 5% CO_2_ at 37°C.

An LSR shRNA (targeting sequences of 5′-TGCTGAGCTACTCCTGTCAACGTCTCGTTTTGGCCACTGACTGACGAGACGTTCAGGAGTAGCT-3′) was cloned into a pcDNA6.2-GW/EmGFP-miR (Invitrogen, Carlsbad, CA, USA). This expression vector used plenti6.3/V5 DEST vectors. After amplification and DNA sequencing confirmation, this vector or negative control (NC) vector was used to generate lentiviruses in HEK-293 cells. These lentiviruses were used to infect HPDE6-C7 cells and named as LSR-knockdown (LSR-KD) cells or NC cells. In addition, a lentiviral vector carrying human LSR cDNA or vector only was used to infect HPDE6-C7 cells with LSR overexpression (LSR-OE) in cells. The efficiency of LSR-KD or LSR-OE was confirmed using RT-qPCR and Western blot. Moreover, to determine the effects of LSR on HPDE6-C7 cells in the HTG environment, cells were treated with 2 mM palmitic acid (PA) (Sigma, USA) for 24 h prior to use for subsequent experiments.

### 2.7. Western Blot Analysis

Cells were homogenized in RIPA lysis buffer (Solarbio, China), and protein concentrations were determined using the BCA protein assay kit (Beyotime, China). Total samples (20 *μ*g) were separated using 10% SDS-PAGE and then transferred to a PVDF membrane. After blocking with 5% nonfat milk for 1 h, samples were incubated overnight at 4°C with primary antibodies against *β*-actin (1 : 3000 dilution, Affinity, USA), LSR (1 : 3000 dilution, Abcam, USA), TRIC (1 : 3000 dilution, Abcam, USA), ZO-1 (1 : 800 dilution, CST, USA), occludin (1 : 1000 dilution, Abcam, USA), and claudin-1 (1 : 2000 dilution, Abcam, USA). Then, the membrane was washed three times using Tris-buffered saline with Tween-20 (TBST) buffer and incubated for 60 min at room temperature with peroxidase-conjugated AffiniPure Goat Anti-Rabbit secondary antibody (CST, USA). Protein bands were visualized using the Odyssey infrared laser imaging system (Licor, USA) and were quantified through Quantity One software (Bio-Rad Laboratories).

### 2.8. Cell Monolayer Permeability

The permeability of HPDE6-C7 cells grown on Transwell filters (0.4 m pore size; BD Biosciences) was assessed through the passage of FITC-Dextran. Briefly, HPDE6-C7 cells were seeded into the upper chamber to form a confluent cell monolayer. Then, FITC-Dextran was added to the upper chamber. Finally, the medium at the bottom of the well was collected and the appearance of fluorescence was monitored at 490 nm excitation and 520 nm emission.

### 2.9. Statistical Analyses

All experiments were repeated at least three times and all data were expressed as mean ± standard deviation (mean ± SD), and categorical variables were expressed as mean (ratio). Data were analyzed using the statistical software SPSS 20.0 (IBM SPSS, Armonk, NY, USA). Statistical significance was determined using Student's *t*-test or analysis of variance (ANOVA) as appropriate, and the nonparametric variance test (Kruskal-Wallis test) was used to compare data with nonnormal distribution. One-way analysis of variance and the Bonferroni post hoc test were used to determine differences among multiple groups. Categorical variables were evaluated using the *χ*^2^ test, and Fisher's exact test was used when the number of observations was less than five. *P* < 0.05 indicated a statistical difference.

## 3. Results

### 3.1. Weights and Serum TG Values

The rats in the high-fat group weighed significantly more than those in the normal diet group (210.50 ± 14.38 vs. 253.30 ± 20.39 g, *P* < 0.05). Serum collected from the rats in the high-fat diet group was turbid, while that in the normal diet group was clear. The serum TG levels in rats in the HTG and HTGAP groups were significantly higher than the corresponding levels in the C group and AP group (715.88 ± 33.22 vs. 243.65 ± 23.26 *μ*mol/L and 693.25 ± 35.97 vs. 263.70 ± 52.82 (*P* < 0.05), respectively) (Table 1).

### 3.2. Macroscopic View and Histopathological Analysis of the Pancreas

**Figure 2:**
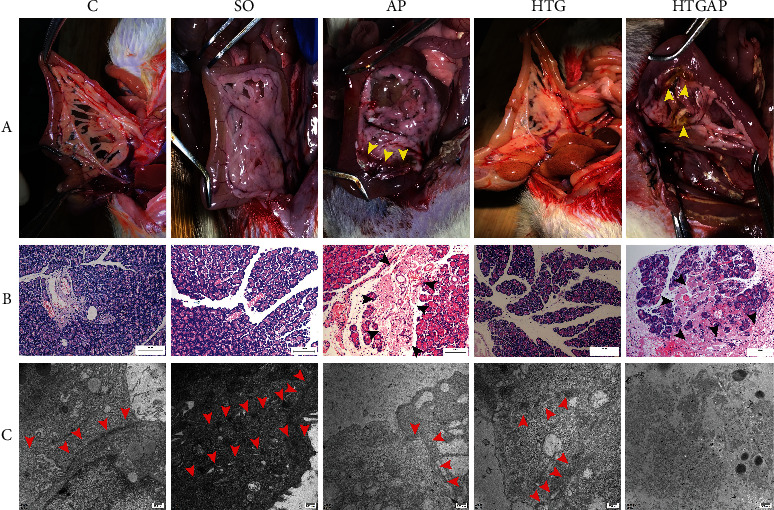
Damage to the pancreas and tight junction in rats. (a) Macroscopic view of the pancreas in different groups. The yellow arrows indicated hemorrhage and necrosis of the pancreas. (b) Pancreatic pathology in different groups (200×). The black arrows indicate pancreatic necrosis. (c) The tight junction of pancreatic ductal cells under an electron microscope (×40,000). The red arrows indicate the cell-cell conjunctions of the pancreatic ductal epithelia.

Edema, hemorrhage, and necrosis were observed in the macroscopic view of the pancreas in AP and HTGAP groups (Figure 2(a)). Furthermore, edema, inflammation, and necrosis were observed in AP and HTGAP groups (Figure 2(b)). The pathological score of the pancreas in the AP group was markedly higher than that in the SO group, whereas the HTGAP group showed more severe pancreatic injury compared with the HTG group. However, no significant difference was observed between the scores of HTGAP and AP groups (Table 1).

### 3.3. Effects of HTG on the Expression of TJ Proteins in Pancreatic Tissues

**Figure 3:**
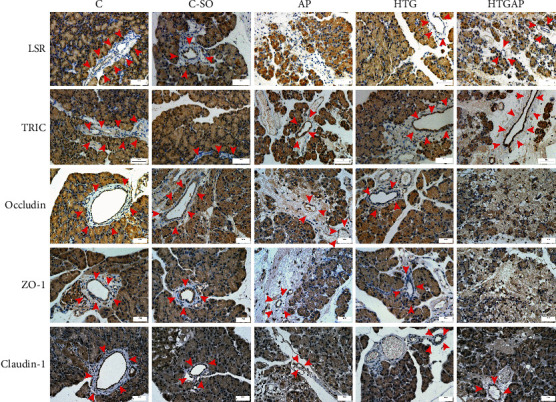
Immunohistochemical staining of tight junction proteins. Expressions of LSR, TRIC, ZO-1, occludin, and claudin-1 in the pancreatic tissues of rats (200x). Red arrows indicate the pancreatic duct.

The expression levels of TJ proteins in the inflammatory and necrotic regions were decreased or disappeared. The expression levels of LSR in the HTG group were significantly lower than those in the C group. The expression levels of LSR, TRIC, occludin, ZO-1, claudin-1, and claudin-7 in AP and HTGAP groups were lower than those in the corresponding control group. The expression levels of LSR, TRIC, ZO-1, and claudin-1 in the HTGAP group were lower than those in the AP group. However, a significant difference was found in the LSR expression (Figure 3, Table 1).

### 3.4. HTG Impaired TJ Structure of Biliary-Pancreatic Ductal Epithelia

In the HTG group, there were few cell-cell TJs in the biliary-pancreatic ductal epithelia, and the intercellular space was wide. However, the TJ count in the biliary-pancreatic ductal epithelial cells in the AP group was remarkably decreased, and the intercellular space in this group was wider than that in the group. Moreover, widespread cellular necrosis was observed in the HTGAP group. However, no cell-cell conjunctions were found in this group (Figure 2(c)).

### 3.5. Effects of PA on the Expression of TJ Proteins and Impairment of the Cellular Barrier of HPDE6-C7 Cells

**Figure 4:**
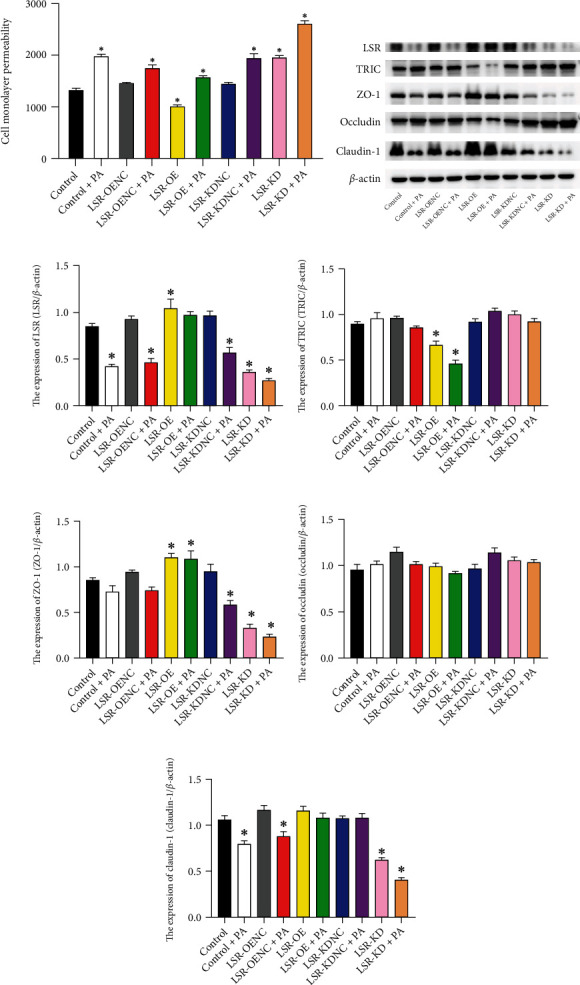
Monolayer permeability and tight junction proteins of HPDE6-C7 cells. (a) Fluorescence values of FITC-Dextran in HPDE6-C7 cells. (b) Western blot expressions of LSR, TRIC, ZO-1, occludin, and claudin-1 in HPDE6-C7 cells. (c) Expression levels of LSR in HPDE6-C7 cells. (d): Expression levels of TRIC in HPDE6-C7 cells. (e) Expression levels of ZO-1 in HPDE6-C7 cells. (f) Expression levels of occludin in HPDE6-C7 cells. (g) Expression levels of claudin-1 in HPDE6-C7 cells. ^∗^vs. control group (*P* <0.05). Monolayer permeability and Western blot analyses were tested at least three times.

In this study, we found that PA downregulated the expression levels of LSR, ZO-1, and claudin-1; upregulated the TRIC expression; and increased the monolayer permeability of HPDE6-C7 cells (Figure 4).

### 3.6. Alteration of the Expression of TJ Proteins and Monolayer Permeability of HPDE Cells due to Regulation of LSR

LSR-OE upregulated the expression levels of ZO-1 and claudin-1, downregulated the expression levels of TRIC and occludin, and alleviated the cellular barrier impairment induced by PA. In turn, LSR-KD downregulated the expression levels of ZO-1 and claudin-1, upregulated the expression levels of TRIC and occludin, and aggravated the cellular barrier impairment induced by PA.

## 4. Discussion

In this study, we investigated the effects of LSR on TJs of PDECs in HTGAP. The findings of the study showed that the level of TG in the serum of rats in the high-fat diet group was significantly higher than that in the normal diet group. The rats in the high-fat diet group weighed significantly more than those in the normal diet group, and the increased weight in the high-fat diet group may have been a risk factor for HTGP. Obesity may contribute to increased AP incidence and worsened severity of AP by release of lipolytic UFA [10]. In particular, visceral abdominal adiposity has the greatest effect on AP [15]. In terms of the pathological analysis, the HTGAP group showed more severe pancreatic injury compared with the HTG group. Furthermore, this study indicated that HTG decreased the TJ protein expression and aggravated pancreatic injury in AP. The regulation of LSR could change the effect of HTG on PDEC barrier function by regulating TJ proteins.

Our animal study showed that the pathological damage of the pancreas in the HTGAP group was more serious than that in the non-high-fat group. Animal studies have shown that the pathological damage of the pancreas in high-fat diet AP models is more serious than that in the non-high-fat AP model [16, 17]. This study found that HTG could aggravate the pathological damage of the pancreas in case of AP, especially in the aspect of pancreatic inflammation. This finding is consistent with the previous study [18]. Furthermore, PA is lipotoxic to pancreatic cells. At present, studies on lipid toxicity of PA mostly focus on pancreatic beta cells and acinar cells [19, 20]. Studies have shown that PA can induce endoplasmic reticulum stress [19], increase activation of NF-*κ*B [21] in acinar cells, and induce apoptosis of pancreatic beta cells and mitochondrial dysfunction [22]. Studies have shown that HTG or PA impairs barrier functions, such as the blood-brain barrier [23] and intestinal barrier [16, 17, 24] by disrupting the TJ integrity.

The TJ is a multifunctional macromolecular complex composed of a variety of proteins and molecules, which is widely present at the top of all connections between epithelial or endothelial cells and has a barrier function [25, 26]. It is mainly composed of transmembrane proteins, occludin, claudin family, adhesion molecules, cytoplasmic proteins, zonula occludens (ZO), and cingulin. TJ consists of bTJ and tTJ. The bTJ is distributed in the lateral membrane between two cells, while tTJ is at the top of where three polygonal cells or epithelial cells meet.

TRIC is the first tTJ protein reported and named by Ikenouchi et al. in 2005 [27]. TRIC is located at the top of the three cells and is one of the most important structures in maintaining the epithelial barrier. When under inflammatory condition, TRIC shifts to the bTJ [28, 29]. LSR, also called angulin-1, is a single transmembrane protein located at the center of the tTJ. It is the core of the angulin protein family. Its main function is to regulate the expression of TRIC, adding TRIC shift caused by various factors, to maintain the structure and function of tTJ [30, 31, 32]. After the LSR-KD, the expression of TRIC on the membrane also disappeared. Fallon et al. In 1995, we stimulated rat pancreas samples with high-dose cerulein. After 30 min, rat PDECs changed into clusters and cell permeability of ZO-1 increased [33]. In another study, Schmitt et al. confirmed that the stimulus with large doses of cerulein resulted in the disintegration of occludin and claudin 1, appearance of lobules within ductal cells, appearance of the acinar cell membrane, and dense dyeing with discontinuous irregular ZO-1 [34]. In this study, the expression levels of claudin-1 and ZO-1 in pancreatic tissues were significantly decreased compared with those before modeling. Currently, only a few reports have investigated the changes in TJ proteins in the pancreatic tissue of HTG/HTGAP animal models.

LSR is located on the cell membrane of the pancreatic duct in normal individuals and in well-differentiated pancreatic duct adenocarcinoma and is slightly expressed in the cytoplasm of poorly differentiated pancreatic duct adenocarcinoma cells [35]. Currently, research on LSR focuses primarily on neoplastic diseases rather than on inflammatory diseases. LSR deficiency affects the malignancy of a variety of tumors, including bladder cancer, colon cancer, endometrial cancer, and head and neck cancer [35–37]. In addition to the localization of LSR in tTJs, some studies have confirmed that LSR is also localized in the nucleus [8, 38]. Reaves et al. [8] found that LSR was expressed in the cell membrane, cytoplasm, and nucleus in a variety of cells, and the approval of LSR suggested a poor prognosis of breast cancer. Kyuno et al. [39] found that LSR knockout can increase the migration, invasion, and proliferation of human pancreatic cancer cells and reduce cell barrier function. Takumi et al. [31] found that after the deletion of LSR in human endometrial cancer cells, the TRIC levels disappeared in the cells; however, LSR expression remained unchanged after the deletion of LSR. LSR can be seen to have a regulatory effect on TRIC. In conclusion, LSR has a regulatory effect on other TJ proteins. The results of this study showed that LSR could upregulate the bTJ proteins ZO-1 and claudin-1 in HPDE6-C7 cells and reduce the permeability of monolayer cells, whereas LSR-KD could downregulate these proteins and increase cell permeability. It was confirmed that LSR has a regulatory effect on the bTJ protein, which is important to maintain cell barrier function.

TRIC and LSR are major members of the tTJ and are expressed in various tissues. Studies have shown that TRIC is localized in normal human pancreatic acinar and ductal cells but not in pancreatic islet cells. Moreover, although TRIC is overexpressed in well-differentiated pancreatic duct adenocarcinoma, it is weakly expressed in poorly differentiated adenocarcinoma [40]. In vitro studies have shown that after LSR knockout, normal PDECs and endometrial cells of the epithelial barrier are damaged, and there is a significant increase in the migration, invasion, and proliferation of human pancreatic and endometrial cancer cells [39, 41]. After treatment with EGF or TGF-*β*, the expression of LSR can increase, and LSR and TRIC can be induced from tTJ positioning to bTJ [39]. However, most studies on TRIC and LSR are related to tumor diseases. Other innovative findings of the research are that PA downregulated the LSR, ZO-1, and claudin-1 expressions, upregulated TRIC expression, and increased the permeability of HPDE6-C7 cells.

The current limitation of our research concerns the interrelation of LSR and TRIC. PA can impair the intestinal barrier [16, 17, 24] by disrupting the integrity of the TJ. However, HTG also has other metabolites, including FFAs, that may play a role in the increase in PA. Therefore, deeper research into the specific regulation of the TJ protein between pancreatic ductal epithelial cells in HTGAP is warranted.

## 5. Conclusion

In conclusion, HTG impairs the function of PDMB, probably by downregulating the TJ proteins in PDECs. Furthermore, the regulation of LSR may change the effect of HTG on the cell barrier function by regulating TJ proteins.

## Figures and Tables

**Table 1:** The damage of the pancreas in AP rats and the expression of tight junction proteins in pancreas.

Groups	*n*	TG (*μ*mol/L)	Histological scores	LSR	TRIC	ZO-1	Occludin	Claudin-1
C	8	243.65 ± 23.26	0.63 ± 0.52	0.216 ± 0.010	0.279 ± 0.027	0.264 ± 0.029	0.254 ± 0.026	0.310 ± 0.023
SO	8	254.19 ± 30.44	0.50 ± 0.54	0.219 ± 0.010	0.273 ± 0.021	0.265 ± 0.013	0.255 ± 0.024	0.301 ± 0.013
AP	10	263.70 ± 52.82	6.30 ± 0.95^a^	0.124 ± 0.011^a^	0.184 ± 0.019^a^	0.183 ± 0.021^a^	0.184 ± 0.016^a^	0.225 ± 0.015^a^
HTG	10	715.88 ± 33.22^ab^	0.80 ± 0.63	0.138 ± 0.016^ab^	0.215 ± 0.016^ab^	0.259 ± 0.023	0.239 ± 0.017	0.292 ± 0.020
HTGAP	10	693.25 ± 35.97^ab^	7.60 ± 0.70^ab^	0.118 ± 0.018^ab^	0.173 ± 0.013^a^	0.167 ± 0.011^a^	0.173 ± 0.015^a^	0.211 ± 0.011^a^

Serum TG level, histological scores of pancreatic tissues [14] and the AOD values of LSR, TRIC, ZO-1, occludin, and claudin-1 in pancreatic tissues of different groups. ^a^vs. C group (*P* < 0.05); ^b^vs. AP group (*P* <0.05).

## Data Availability

All data included in this study are included in the article.

## Abbreviations

AP: Acute pancreatitis
HTG: Hypertriglyceridemia
HTGAP: Hypertriglyceridemic acute pancreatitis
KD: Knockdown
LSR: Lipolysis-stimulated lipoprotein receptor
NC: Negative control
OE: Overexpression
PA: Palmitic acid
PDEC: Pancreatic ductal epithelial cells
PDMB: Pancreatic ductal mucosal barrier
SO: Sham-operative
TG: Triglyceride
TJ: Tight junction
TRIC: Tricellulin.
